# Physico-Chemical and Nutritional Properties of Chia Seeds from Latin American Countries

**DOI:** 10.3390/foods12163013

**Published:** 2023-08-10

**Authors:** Natalia Vera-Cespedes, Loreto A. Muñoz, Miguel Ángel Rincón, Claudia M. Haros

**Affiliations:** 1 Instituto de Agroquímica y Tecnología de Alimentos (IATA), Spanish Council for Scientific Research (CSIC), C/Catedrático Agustín Escardino Benlloch, 7, 46980 Paterna, Valencia, Spain; nati.vcespedes@gmail.com; 2 Laboratorio de Ciencias de los Alimentos, Instituto de Investigación y Postgrado, Facultad de Ciencias de la Salud, Universidad Central de Chile, Santiago 8330546, CP, Chile; 3 Department of Agronomy, Food Technology Division, University of Almería, La Cañada de San Urbano s/n, 04120 Almería, Spain; marincer@inta.uchile.cl; 4 Institute of Nutrition and Food Technology, University of Chile, El Líbano 5524, Macul, Santiago 7830490, CP, Chile

**Keywords:** *Salvia hispanica* L., physical properties, seed composition, amino acid profile, fatty acid profile, mineral composition, phytic acid

## Abstract

In the last few decades, chia (*Salvia hispanica* L.) cultivation has expanded around the world, and the seeds have become well known due to their rich composition of nutrients and bioactive compounds. The aim of this work was to evaluate the physical, chemical, and nutritional profile of eight types of chia seeds grown in different Latin-American countries (Argentina, Bolivia, Chile, Ecuador, Mexico, Paraguay, and Peru). The results showed that several nutritional parameters of the seeds, such as the protein content and amino acid profile, dietary fiber content, lipid content, mineral composition, and presence of phytate, depend on the location in which they were grown. Other parameters, such as ash content, fatty acid profile, or various physical parameters, were uniform across locations (except for color parameters). The results support the notion that the nutritional characteristics of seeds are determined by the seeds’ origin, and further analysis is needed to determine the exact mechanisms that control the changes in the seed nutritional properties of chia seeds.

## 1. Introduction

Chia (*Salvia hispanica* L.) is an oilseed plant that belongs to the *Lamiaceae* family. It originated in the region of Mexico and Guatemala and was cultivated by Mayans and Aztecs around 3500 BC. The seeds are a natural source of α-linolenic acid (ALA, 18:3 n-3), an omega-3 polyunsaturated fatty acid (omega-3 PUFA), as well as dietary fiber, proteins, natural antioxidants, vitamins, and minerals [1]. Chia seeds contain the highest known percentage of ALA among plant sources [2]. ALA is an important fatty acid associated with certain physiological functions. Chia’s high content of dietary fiber ranges from 34 to 40 g per 100 g of seeds. This amount of dietary fiber meets the daily recommendations of the EFSA and the American Dietetic Association after an intake of around 63–74 g of seeds [3,4]. Moreover, chia seeds are a good source of proteins, containing between 19 and 23 g per 100 g of seeds, which is higher than the protein content in most utilized seeds [5]. In addition, the presence of minerals such as calcium, potassium, and magnesium, along with the presence of vitamins and antioxidants compounds, makes this seed very interesting from a nutritional point of view [6]. In a report from the Institute of Food Technology, consumers defined a healthy food as a food that is high in nutrients and/or healthy components, such as phenolic compounds and vitamins, as well as in fiber and proteins. In this context, chia seeds are a promising source of these components, among others [7].

Currently, chia seeds are considered a healthy ingredient in the framework of a balanced diet, which is why their cultivation has expanded to many countries [8]. Chia grows naturally in tropical and subtropical environments in frost-free areas and in regions with annual frosts, from sea level to 2500 m [9]. Chia was originally a short-day flowering species; the area where it could produce seeds was therefore limited to a restricted range of latitudes; however, new genotypes have been introduced to extend the range to other temperature areas and regions [10]. These plants prefer sandy, well-drained soils with moderate salinity and a pH ranging from 6 to 8.5 [11]. In this sense, it has been found that the chia seed composition and physical characteristics of chia seeds can vary according to the geographical location and climatic conditions and these environmental parameters can influence the profile and concentration of nutrients available in the seeds [12].

This is also the case in the vast majority of crops; nevertheless, chia has some restrictions in terms of its chemical composition, with minimal contents determined for lipids, dietary fiber, and proteins because of its status as a novel food in the European Union [13], including as a source of oil [14]. According to a marketing study by the Centre for the Promotion of Imports from developing countries (CBI), Ministry of Foreign Affairs (The Netherlands) published a few years ago, the commercial production of chia was low and concentrated in specific areas [15]. Nowadays, chia is cultivated in many countries such as Mexico, Argentina, Australia, and Ecuador, as well as in Europe, although the seed quality is not the same as those produced in Latin America. In this context, the aim of this work was to characterize and comparatively analyze some physico-chemical and nutritional properties of chia seeds grown in different Latin American countries.

## 2. Materials and Methods

### 2.1. Materials

Chia seeds produced in Latin American countries were obtained from local markets. The following is a list of the countries from which seeds were obtained and seeds’ origin within that country: (i) Bolivia: two phenotypes, white and dark seeds, produced in Tarija and commercialized by Benexia (Functional Products Trending S.A.); (ii) Chile: seeds produced in San Vicente de Tagua Tagua by SPS Foods (South Pacific Seeds); (iii) Argentina: seeds produced in Salta and sold by Villares S.A.C.; (iv) Ecuador: seeds produced in Latacunga and commercialized by Inca’s Treasures; (v) Peru: seeds produced in Chorrillos and marketed by Naturandes Company, Copiapo, Chile; (vi) Mexico: seeds produced in Jalisco and commercialized by EcoPan Organics Trends; and (vii) Paraguay: seeds produced in San Pedro and sold by Natural Factor Company, Monroe, WA, USA.

Unless otherwise stated, all solvents and reagents used in this work were purchased from Merck and Sigma-Aldrich (Darmstadt, Germany).

### 2.2. Physical Properties of Chia Seeds from Different Origins

The seeds’ morphology was analyzed using a stereomicroscope, Leica model S8 APO, equipped with a digital camera, Leica model MC 170 HD. To determine the average size of the seeds, samples of 100 units were randomly selected and positioned in two different orientations, vertically and horizontally, in a slide with double contact tape [16], and images from the different positions were acquired. The images were examined by image analysis using ImageJ software [17]. Firstly, the images were binarized (black and white); then, the three dimensions, length (L), width (W), and thickness (T), were determined. The geometric diameter (Dg) and the sphericity (ɸ) were calculated using Equations (1) and (2), respectively [18]:

Dg = (LWT) ^(1/3)^ (1)

ɸ = (LWT) ^(1/3)^/L × 100 (2)

where L is the length, W is the width, and T is the thickness, all of them expressed in mm.

The surface area (S) expressed in mm^2^, was determined using Equation (3):

S = π Dg^2^ (3)

where Dg corresponds to geometric diameter.

The average bulk density (*ρb*) was obtained by filling a 100 mL test tube with seeds and weighing the content, according to Ixtaina et al. [19]. The true density (*ρt*) was determined with the displacement method in a pycnometer using hexane as the liquid. The absorption of hexane was considered negligible due to the short duration of the procedure. The porosity of the bulk (ε), defined as the fraction of space not occupied by the grain, was calculated as a percentage of porosity by using Equation (4), and the volume of one seed (V) measured in mm^3^ was determined according to Equation (5).

(4) $$\varepsilon =\left(\frac{\rho t-\rho b}{\rho b}\right)\times 100$$

(5) $$V=\left(\frac{m}{\rho t}\right)\times 100$$

The equivalent diameter (De) was determined as the diameter of a sphere having the same volume as the seed (Equation (6)).

(6) $$\mathrm{De}=({\frac{6V}{\pi })}^{1/3}$$

The weight of 1000 seeds (W_1000_) was determined by analyzing five samples of 100 seeds from each country. Each sample was weighed in an electronic balance with a 0.0001 g accuracy (model BA2204B, BIOBASE, Shandon, China) and the weight was extrapolated to 1000 seeds.

The color was determined for the seeds and their flours. The seeds were milled, and the color parameters were determined using a chromameter (model Chroma Meter CR-400, Konica Minolta, Tokyo, Japan). The values were expressed in L* (lightness); a* (red to green) and b* (yellow to blue) in the CIELab system.

### 2.3. Proximate Chemical Composition

The proximate composition analysis measuring moisture, proteins, lipids, and ash, was performed in triplicate using AACC methods, and the results were reported as g/100 g on a dry basis [20]. The moisture was determined by placing the seeds in an oven (model, Biobase, China) at 105 °C until they reached a constant weight; the nitrogen content was determined using the Kjeldahl method, in which the protein was calculated using a nitrogen conversion factor of 6.25, the lipid fraction was extracted with hexane under reflux conditions using the Soxhlet technique (Soxtec 2050, FOSS); and the ash content was obtained by incineration in a muffle furnace at 600 °C according to official methods 08-03 [21]. The total (TDF), soluble (SDF), and insoluble (IDF) dietary fiber content, were determined by the total dietary fiber assay procedure of the AOAC method 991.43, based on an enzymatic and gravimetric method by using a K-TDFR kit, Megazyme, Wicklow, Ireland [22].

### 2.4. Amino Acid Profile

The amino acid profiles were determined using the adapted method of the European Commission. The contents of cysteine and methionine were determined according to the oxidative hydrolysis, amino acid analyzer with the ninhydrin method [23,24]; the tryptophan was measured according to alkaline hydrolysis and quantification by HPLC techniques [23,24]; and the others amino acids were determined using the acid hydrolysis, amino acid analyzer with the ninhydrin method using the same standards.

### 2.5. Oil Extraction and Fatty Acids Profile

The oil extraction was carried out according to the Folch extraction method [25]. Aliquots of 5 g of ground seeds were weighed and a mixture of chloroform:methanol 2:1 *v*/*v*) was added (20:1 *v*/*w*). The mixture was stirred and then vacuum filtered to remove the defatted seed meal. Then, the solvent was removed from the filtrate in a rotary evaporator at 40 °C, and the resulting chia oil was kept at −20 °C under an inert nitrogen atmosphere until further analysis.

The fatty acid profile was determined by gas–liquid chromatography coupled with a flame ionization detection (GC-FID) described by Rincón-Cervera et al. [26] using the Agilent 6890N equipment and a 7683B autosampler (Agilent Technologies, Santa Clara, CA, USA). Fatty acid identification was carried out according to the respective retention times through the capillary column Supelco SP-2560 (100 m × 0.25 mm × 0.2 μm film) (Sigma-Aldrich, St. Louis, MO, USA) compared to analytical standards (37 FAME Mix components from Supelco, Sigma-Aldrich, St. Louis, MO, USA).

### 2.6. Mineral Composition and Phytic Acid Determination

Minerals were measured with a flame atomic absorption spectrometer at the Analysis of Soils, Plants and Water Service in the Institute of Agricultural Sciences, Madrid (Spain).

The samples were previously digested by means of HNO_3_ and H_2_O_2_ attack by irradiating at 800 W (15 min at 180 °C) in a Microwave Accelerated Reaction System (MARS, Charlotte, NC, USA).

Phytic acid (*myo*-inositol 1,2,3,4,5,6-hexakisphosphate, Ins*P*_6_) was measured as phosphorus released by the action of phytase and alkaline phosphatase determined through a spectrophotometric method using a commercial kit (K-Phyt 07/11 Megazyme, Wicklow, Ireland). Samples were analyzed in triplicate.

### 2.7. Statistical Analysis

All analyses were performed in triplicate and results were reported as mean value ± standard deviation. One-way ANOVA was used to compare means values, and significant differences (*p* < 0.05) were calculated with Tukey´s post hoc test. All tests were performed using the software Statgraphics Centurion XV.I.

## 3. Results and Discussion

### 3.1. Physical Properties and Morphology of Chia Seeds from Different Origins

**Figure 1:**
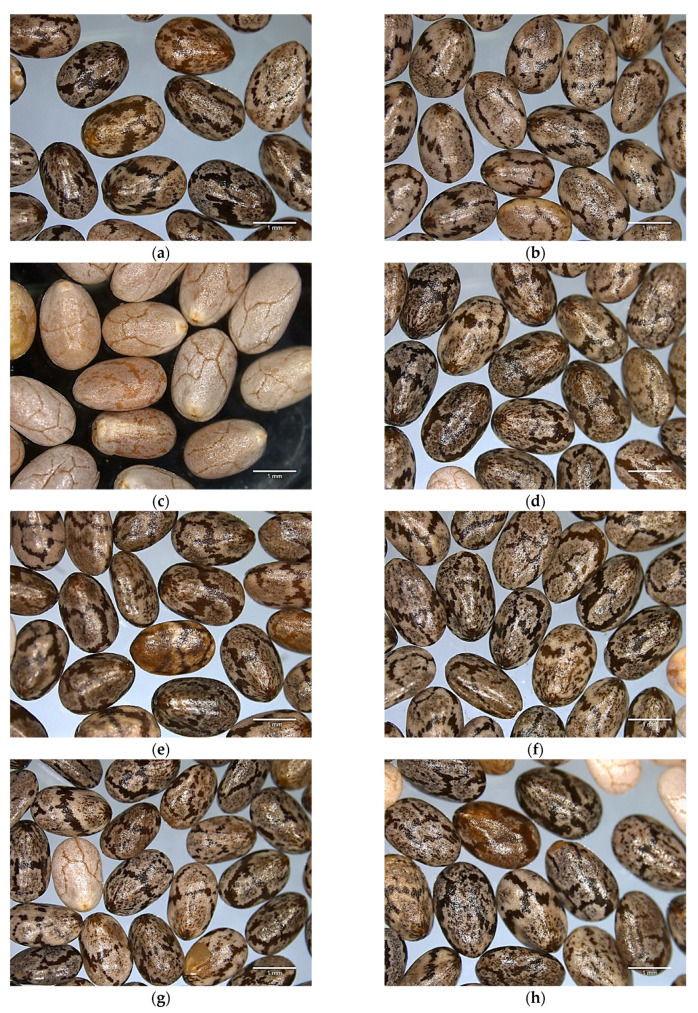
Morphologic characteristics of commercial chia seeds from different origins. (**a**) Chia seed from Argentina; (**b**) Chile; (**c**) Bolivia (white seed); (**d**) Bolivia (dark seed); (**e**) Ecuador; (**f**) Mexico; (**g**) Paraguay; (**h**) Peru.

The physical properties and morphology of chia seeds from different origins are summarized in Table 1 and Figure 1, respectively, while the colors of seeds and whole ground seeds are summarized in Table 2. The morphologic features of the seeds are an important issue, useful to the design of crop production and harvest tools, as well as to know how to store them [27]. Significant differences (*p* < 0.05) were found for the seeds in terms of size, color, and morphology regarding their country of origin. The longitudinal dimension (L) ranged from 1.867 ± 0.119 mm (Ecuador) to 2.059 ± 0.104 mm (Paraguay), while the width dimension ranged from 1.189 ± 0.103 mm (Ecuador) to 1.370 ± 0.093 mm (Chile), with the Ecuadorian seed being the smallest and the white seed from Bolivia the biggest. With regard to the surface area, the white seeds also exhibited the highest value at 6.42 ± 0.58 mm^2^. The bulk density of the seeds from the different countries was significantly different, with the highest value seen in seeds from Paraguay. No significant differences were found in true density between seeds from Bolivia, Paraguay, Chile, and Perú, but significant differences were found in seeds from Argentina and Mexico. Seed porosity characterizes the open structure of the material, which is the fraction of empty volume [28]. In this study, porosity was lower in Argentinian and Paraguayan seeds and higher in Bolivian, Chilean, Peruvian, and Paraguayan seeds, without significante differences between the groups.

In general, all the seeds showed an obovoidal to ellipsoidal shape with a rounded base and apex. This made the % sphericity lower than 1, falling between 0.67 ± 0.02% (Argentina and Peru) and 0.73% (Chile). In agreement with Muñoz, Cobos, Diaz and Aguilera [16], the seeds displayed an oval flattened shape and ranged in color from dark coffee to beige. In addition, the color of the seed coat varied from black, grey, and black or dark spotted to white, as it can be seen in Figure 1. These observations are also in agreement with Knez Hrnčič et al. [29]. The main pigments associated with the seed color, such as carotenoids and chlorophyll have been identified, as described by Amato et al. [30]. Seeds from Mexico showed a narrower seed coat with more brown stretch marks than seeds from Ecuador, which showed less and wider stretch marks, while Chilean seeds had a more uniform colored coat with more translucent streaks. Finally, the white seeds from Bolivia showed small and fine darker brown indentations on the seed coat. 

Table 2 shows the color in terms of L*, a*, and b* of whole and ground seeds and Table 3 shows the comparison of the total difference (*ΔΕ*) between seeds and whole ground seeds. Overall, the whole ground seeds showed significant differences among them (*p* < 0.05). The highest L* value was found in the white seeds from Bolivia, as expected, followed by the Chilean seeds, while the lowest value was found in the seeds from Peru. According to Mokrzycki and Tatol [31], when *ΔΕ* > 3.5, a standard observer sees the differences between two colors and can difference them. In the case of the white seeds and seeds from Chile, when compared to the rest of the seeds, all Δ*Ε* values were greater than 3.5, which implies that the differences could be detected with the naked eye. However, among the other seeds, it was not possible to detect these differences. The same behavior was observed in the whole ground seeds.

In general terms, the color of the whole ground seeds, which are used to produce food products, can influence the acceptability of the end product to the consumer [32].

### 3.2. Proximate Chemical Composition of Seeds

The proximate composition of the seeds is shown in Table 4. In general, the moisture of the chia seeds from the different countries ranged between 7.09 ± 0.11 and 9.15 ± 0.21 g/100 g.

The protein concentration varied between 17.7 ± 0.1 g/100 g of seeds (Ecuadorian chia) and 24.3 ± 0.0 g/100 g of seeds (white Bolivian chia) g/100 g of seeds in dry matter (Table 4). The levels of protein in chia seeds from different origins were shown to be in the following descending order: dark Bolivian (dark) > Chile > Bolivian (white) > Paraguay > Mexico > Peru > Argentina > Ecuador (Table 4). In general, the values were higher than those reported for seeds from subtropical ecosystems in South America, such as Brazil; however, this paper’s results were in concordance with past results for Ecuadorian chia seeds [12,33,34].

This variation can be attributed to agricultural factors, such as growing region, stage of plant development, temperature, soil, light, and genotype, as previously reported by Ayerza and Coates [34] and Porras-Loaiza et al. [35]. In the current study, all the chia seeds investigated showed a higher protein content than other seeds such as quinoa (ranging between 13 and 16.7%), amaranth (ranging from 12.5 to 16%), and safflower (12.6%), and even showed higher protein levels compared to other oilseeds such as flaxseed (17.9%), sunflower seed (19.3%), and sesame seed (17.7%) [36,37,38,39]. 

Regarding lipids, Chilean seeds showed the highest amount, with 34.93 ± 0.65 g/100 g, followed by seeds from Paraguay, with 34.51 ± 0.20 g/100 g and Bolivia (dark seeds), with 32.13 ± 0.89 g/100 g, which were significantly higher than seeds from the other countries screened in this study. Lipid content is normally associated with climatic conditions. While lower temperatures increased the content of lipids and the level of fatty acid unsaturation, high temperatures lead to a decrease in lipid content [33]. This paper´s results were similar to those previously reported by Shen et al. [40] who analyzed chia seeds from Mexico and they were consistent with data reported by Ayerza and Coates [12] from chia seeds from Ecuador.

In general, the total dietary fiber ranged from 25.97 to 16.79 g/100 g seed. According to the EFSA and the WHO/FDA, the daily recommendation for dietary fiber intake for adults is in the range of 20 to 35 g/day, which makes the chia seeds analyzed in this work an excellent source of fiber [41,42]. The highest amount of dietary fiber was found in seeds from Peru with 25.97 ± 0.35 g/100 g, followed by the seeds from Ecuador with 22.42 ± 0.40 g/100 g, and Chile, with 21.05 g/100 g seeds (Table 5). In all cases, the amount of dietary fiber was higher than that from grains and seeds such as quinoa, amaranth, and flaxseed [1].

Differences found regarding the contents of lipids, proteins, dietary fiber, and minerals, among others, might be attributed to the different seeds’ genotypes or to environmental and climatic factors, which are related to the geographical location.

### 3.3. Amino Acid Profile

**Figure 2:**
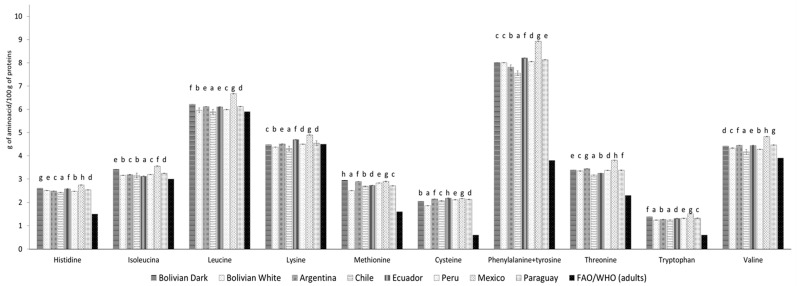
Composition of amino acids (mg/g protein) based on the FAO/WHO/UNU (Food and Agriculture Organization/World Health Organization/United Nations University) standard adults reference pattern (g/100 g protein) [44]. Values are expressed as mean ± standard deviation (n = 3). Bars followed by the same letter are not significantly different at 95% confidence level.

Their high-protein content makes chia seeds attractive from a nutritional point of view. The nutritional contribution of vegetable proteins to the maintenance of human health depends on their biological quality, given by the presence of all the essential amino acids. In this sense, Table 6 shows the amino acid composition of chia seeds from different origins, both essential and not essential. According to the results, the amount of essential amino acids (EAAs) in chia seeds from different origins did not follow the same trend found with the protein content. The levels were found to be, which was in the following descending order: Bolivia (dark) > Mexico > Bolivia (white) > Chile > Paraguay > Argentina > Peru > Ecuador (Table 6), showing that protein quality is regardless was independent of protein content, as is shown in Figure 2. In this sense, comparing the essential amino acids based on the WHO/FAO/UNU [44] standard adults reference pattern (g/100 g protein) to the chia proteins levels in general, showed that all the chia studied presented an adequate amino acid profile, with the exception of lysine in the chia from Chile and white Bolivian seeds. These seeds contained 4.31 ± 0.10 and 4.37 ± 0.01 g/100 g of proteins, respectively, which was less than the reference level of 4.5 g/100 g of proteins (Figure 2). The highest amount of lysine was present in the Mexican chia protein, followed by the Ecuadorian chia protein (Figure 2). Previous investigations reported that chia seeds contained limiting amino acids such as lysine, as well as leucine and threonine [45]; however, in the current study, limiting amino acids did not appear. This discrepancy could be due to the different varieties, soils, and climatic conditions of the crops, as was reported by Ayerza [46].

On the other hand, the non-essential amino acid profile of the chia seeds included abundant amounts of aspartic acid (1.47–2.25 g/100 g), arginine (1.78–2.62 g/100 g), and glutamic acid (3.17–4.41 g/100 g), as was previously described by other researchers [47,48].

### 3.4. Fatty Acids Profile

The fatty acid profiles of the chia seeds are summarized in Table 7. According to Venskutonis and Kraujalis [37], the fatty acids composition of edible oils determines their nutritional, functional, and technological properties. The main fatty acids available in all the seeds were α-linolenic acid (ALA) with values between 14.91 and 18.35 g/100 g (55.2–65.9% of total lipids), followed by linoleic acid (LA) with values between 4.88 and 5.97 g/100 g (17.8–22.1% of total lipids), and palmitic acid, which ranged between 1.89 and 2.15 g/100 g (6.9–7.8% of total lipids). The proportion of polyunsaturated fatty acids (PUFAs) was about 79.1 to 83.9% of total lipids in chia seeds, which is similar to the previously reported data by several authors [8,40,49], but higher than values found in chia seeds from Africa (Kenya and Uganda), where the amounts of ALA and LA ranged from 45.3 to 57.0% and from 15.9 to 20.3%, respectively [50]. The seeds from Paraguay and Peru showed the highest amount of PUFAs, followed by those from Argentina and Chile. Regarding ALA, the highest content was found in seeds from Chile and Argentina, followed by seeds from Peru and Paraguay. These differences may be attributed to several factors that can influence the biosynthesis of target compounds (i.e., essential fatty acids) such as environment and climate conditions, temperature, soil type, and availability of nutrients, among others [12,51].

The n-6/n-3 PUFA ratio ranged between 1/2.5 and 1/3.7, in agreement with previously reported values by Knez Hrnčič, Ivanovski, Cör and Knez [29], and Shen, Zheng, Jin, Li, Fu, Wang, Guan, and Song [40]. This was due to the high proportion of ALA and n-3 PUFAs present in the chia seed oil. The consumption of foods with low n-6/n-3 PUFA ratios may contribute to lowering the risk of coronary heart disease, hypertension, and metabolic syndrome, among other illnesses [53]; on the contrary, an imbalance of these fatty acids in favor of n-6 PUFA could contribute to the prevalence of atherosclerosis, obesity and diabetes, among others [54,55,56]. Conventional diets in most Western countries are rich in n-6 PUFA, reaching n-6:n-3 PUFA ratios of 20:1. A 5:1 or 4:1 n-6:n-3 PUFA ratio is usually recommended to balance the intake of both types of PUFA. Therefore, the consumption of foods rich in n-3 PUFA such as chia seeds may be a suitable way to increase the dietary proportion of these key nutrients. The values of the n-6/n-3 PUFA ratio in chia seeds oil found in the current work were lower than those from other vegetable oils such as flaxseed, soybean, olive, and canola oils, among others, which is highly desired for a healthy diet [57].

Based on the dietary reference intake (DRI) from the National Academy of Sciences for ALA and LA, chia seed oil could be consumed as a supplement [58].

### 3.5. Mineral Composition

The mineral composition of the chia seeds is shown in Table 8. According to the results, calcium (Ca) was the most abundant macroelement found in the seeds, ranging between 460 and 671 mg/100 g. Seeds from Chile and Peru reached the highest values; phosphorus (P) ranged from 394 to 662 mg/100 g and the highest values were found in Chilean seeds (indicating higher phytates). Magnesium (Mg) ranged between 250 and 322 mg/100 g, and sodium (Na) between 0.8 and 1.4 mg/100 g. Differences in the content of microelements were found in the different samples of chia seeds. These included iron (Fe) (4.2–23.4 mg/100 g), zinc (Zn) (2.7–5.2 mg/100 g), manganese (Mn) (0.9–5.9 mg/100 g), and cupper (Cu) (0.9–1.6 mg/100 mg). Barreto et al. [59] reported a similar value for calcium and higher values for phosphorous, iron, zinc, and copper, whereas the United States Department of Agriculture reported similar values for Ca, Mg, Mn, and Cu and higher values for P, Na, Fe, and Zn [60]. Peruvian chia seeds reached an Fe value of 23.4 mg/100 g, which could be related to the concentration of this mineral in the soil where the plant grew and the plant’s absorption ability. In turn, the concentration of some microminerals, such as Fe and Zn, could vary depending on the characteristics of the region (type of soil, precipitation level) and/or the application of fertilizers [61].

In terms of the dietary references intakes (DRIs), and considering mineral absorption inhibitors are absent, Table 8 shows the contribution to the DRI of chia seeds expressed in % and calculated based on an intake of 15 g/day, which is the maximum recommended intake level of chia seeds by the EFSA NDA Panel [52]. In general, the results showed that the chia seed is a good source of minerals. In this context, the seeds from Chile presented the highest contributions towards the DRIs of Na (6.71%), Ca (10.07%), P (18.05%), Mg (13.78/16.08%), and Cu (14.81/18.23%). The white Bolivian seeds were also provided.

The highest contribution of Zn (6.59/9.06%). In addition, the seeds from Peru showed a significantly higher contribution of Fe (31.91/50.14%), while seeds from Paraguay and Ecuador stood out for their high contributions of Mn (38.80/49.58% and 25.76/32.92, respectively) (man/woman, respectively).

Regarding aluminum (Al), the lowest and highest values were found in seeds from Paraguay (0.25 ± 0.03 g/100 g) and Peru (41.15 ± 0.16 g/100 g), respectively (Table 8), whereas the values in the other seeds ranged between 0.8 and 4.15 g/100 g. According to Bojórquez-Quintal et al. [63], high Al amounts could be attributed to the acid pH of the soil (≤5.5). These soils are characterized by a nutrient deficiency and the presence of metals such as Al, which can have a beneficial effect on plants by stimulating the absorption of nutrients. The effects of Al on humans are still poorly studied; however, the World Health Organization [64] estimates a tolerable intake of 2 mg/day per kg body weight, due to the daily exposure to this metal through foods, cosmetics, etc.). Seeds from Mexico showed the lowest amount of sulfur (S) (166 ± 0.21 g/100 g), whereas the highest amount was found in seeds from Chile (222.8 ± 0.53 g/100 g). These results could be related to the amount of sulfur used during cultivation in order to obtain a higher yield and quality in the production of oilseeds [65]. There is no dietary reference intake (DRI) for sulfur, although the World Health Organization (WHO/OMS) recommends a daily intake of S-containing amino acids estimated at a methionine requirement of 13 mg/day per kg body weight [58,66].

Phytic acid is an organic acid with chelating characteristics that binds di- and trivalent minerals, such as Ca, Mn, Fe, and Zn, among others, reducing their bioavailability in the monogastric animals and human gut [33,67]. In this study, the phytate values obtained ranged between 1.55 and 2.65 g/100 g. White Bolivian chia registered the lowest value at 1.55 g/100 g, while the Chilean and dark Bolivian chia registered the highest values at 2.63 and 2.65 g/100 g, respectively (Table 8). Similar results were obtained from chia seeds by Pereira da Silva, Anunciação, Matyelka, Della Lucia, Martino, and Pinheiro-Sant’Ana [33], and according to the EFSA NDA Panel [52], these values do not represent a safety concern for consumers. 

The inhibitor effect of phytates on the absorption of Ca, Fe, and Zn can be estimated using the molar ratio of phytate/mineral; the ratio of phytate/Ca should be <0.24, phytate/Fe < 1 and phytate/Zn < 15 to present a low inhibition of these minerals´ bioavailability after consuming chia [43,68]. The absorption of these minerals is a key issue regarding the correct functioning of the human body because of their essential role in growth, immunity, etc. In particular, Ca prevents bone fractures and osteoporosis, and it is involved in muscle contraction, blood clotting, nerve impulses, and fluid balance within cells [69]. Fe participates in metabolic processes such as oxygen transport, DNA synthesis, and electron transport, and its deficit could lead to learning and memory problems and anemia, among other issues [70,71,72]. Zn deficiency causes growth retardation and undesired negative effects on the gastrointestinal, central nervous, immune, skeletal, and reproductive systems [73]. The values obtained for the Ins*P*_6_/Ca molar ratios ranged between 0.22 and 0.37; the Ins*P*_6_/Fe molar ratios were 8.0–52.6 and the Ins*P*_6_/Zn molar ratios were 37.1–94.9. Concerning calcium, the molar ratio being lower than the inhibition threshold value indicates no inhibition on its mineral availability, which was found only in seeds from Bolivia (white variety) and Peru. The rest of the chia seeds showed inhibition in this mineral (Table 8). Additionally, the results indicate that after ingestion of any of the studied chia seeds, the bioavailability of Fe and Zn would be strongly inhibited by phytic acid. In this sense, concerning mineral availability, chia should be included—for example, in the form of ground seeds in food formulations that require fermentation, in which the phytic acid is hydrolyzed by the endogenous seed phytase.

## 4. Conclusions

In general, the physico-chemical and nutritional properties of chia seeds from different origins mainly depend on the agricultural, environmental, and climatic conditions in the areas in which they were grown. In this study, all of the seeds displayed an oval flattened shape and the seed coat color varied from black, grey, and black or dark spotted to white according to the variety and origin of the crop. There was a high amount of protein in the chia seeds, with differences between the different countries, which may be due to the different varieties, soils, and climatic conditions. However, the amino acid profiles included a good balance of essential amino acids compared to the amino acid reference pattern for protein for adults. All chia seeds from the different countries exhibited a high dietary fiber content, which meets almost 100% of the daily recommendations. Due to the high concentration of ALA in all of the samples investigated, chia intake could equilibrate the n-6:n-3 PUFA ratio in human diets, which is usually unbalanced in Western diets. Chia seeds were found to be a good source of minerals, but the minerals will not be bioavailable due to the high concentration of phytic acid in the seeds´ composition. In this sense, for chia to be a nutritious source of minerals, it should be included as an ingredient in fermented food formulations in which the phytic acid is hydrolyzed by endogenous phytases, such as in bread products. Regardless of the origin of the crop, chia has been shown to have high nutritional and functional value for the entire population, including in at-risk populations. Finally, this study showed that chia seeds from different Latin American countries are an excellent source of dietary fiber, omega-3 fatty acids, protein, and minerals, and their consumption can help meet the daily requirements for these important nutrients.

## Figures and Tables

**Table 1:** Physical properties of chia seeds from different origins.

Parameter	Units	Bolivian				Argentina		Chile		Ecuador		Peru		Mexico		Paraguay	
		Dark		White													
1000-seed mass	g	1.38	±0.01 e	1.26	±0.02 b	1.26	±0.03 b	1.26	±0.01 b	1.10	±0.02 a	1.31	±0.02 d	1.29	±0.02 d	1.46	±0.01 c
Bulk density	g/m^3^	69.7	±1.1 d	68.2	±0.3 abc	69.4	±0.4 cd	71.2	±0.4 e	68.5	±1.9 bcd	67.0	±0.1 a	67.8	±0.2 ab	72.9	±0.1 f
True Density	g/m^3^	1154	±40 cd	1162	±0 cd	1102	±0 b	1163	±0 d	1110	±0 ab	1147	±0 c	1122	±0 a	1153	±0 cd
Porosity (ε)	%	94.0	±0.5 e	94.1	±0.1 f	93.7	±0.2 b	93.9	±0.2 d	93.83	±0.9 a	94.2	±0.0 g	94.0	±0.1 e	93.7	±0.1 a
Length	mm	1.98	±0.11 b	2.02	±0.10 cd	2.00	±0.12 bd	1.87	±0.09 a	1.87	±0.12 a	2.03	±1.14 cd	2.01	±0.10 bd	2.06	±0.10 c
Width	mm	1.35	±0.13 e	1.35	±0.09 de	1.25	±0.12 b	1.37	±0.09 e	1.19	±0.10 a	1.31	±0.13 cd	1.30	±0.12 c	1.31	±0.08 cd
Thickness	mm	0.957	±0.081 a	1.08	±0.12 c	0.953	±0.078 a	1.01	±0.09 b	0.946	±0.085 a	0.937	±0.091 a	0.973	±0.086 a	1.01	±0.11 b
Equivalent diameter	mm	0.833	±0.002 a	0.828	±0.012 a	0.878	±0.010 c	0.833	±0.016 a	0.867	±0.003 c	0.838	±0.019 ab	0.859	±0.010 bc	0.843	±0.021 ab
Sphericity (Φ)	%	0.691	±0.038 c	0.706	±0.033 d	0.667	±0.024 b	0.734	±0.034 e	0.686	±0.034 ac	0.667	±0.034 b	0.678	±0.028 abc	0.678	±0.036 ab
Surface area	mm^2^	5.86	±0.52 d	6.42	±0.58 e	5.61	±0.63 b	5.93	±0.55 cd	5.15	±0.55 a	5.76	±0.65 bd	5.84	±0.57 d	6.11	±0.55 c
Volume	mm^3^	1.31	±0.01 a	1.30	±0.02 a	1.38	±0.02 c	1.31	±0.03 a	1.36	±0.01 c	1.32	±0.03 ab	1.35	±0.02 bc	1.32	±0.03 ab
Arithmetic mean diameter	mm	1.43	±0.06 d	1.48	±0.06 c	1.40	±0.08 b	1.42	±0.06 bd	1.33	±0.07 a	1.42	±0.08 bd	1.43	±0.07 bd	1.46	±0.06 c
Geometric mean diameter	mm	1.36	±0.06 d	1.43	±0.06 e	1.33	±0.08 b	1.37	±0.06 cd	1.28	±0.07 a	1.35	±0.08 bd	1.36	±0.07 d	1.39	±0.06 c

Mean ± SD (n = 100). Values followed by the same letter in the same row are not significantly different (*p <* 0.05).

**Table 2:** Color parameter of different commercial chia seeds and their whole ground seeds.

Color Parameters		Bolivian				Argentina		Chile		Ecuador		Peru		Mexico		Paraguay	
		Dark		White													
Seeds	L*	39.6	±0.5 c	59.2	±0.9 e	36.3	±0.2 b	42.1	±0.8 d	33.5	±0.6 a	35.7	±0.8 b	36.3	±0.6 b	32.6	±0.5 a
	a*	1.40	±0.26 a	3.27	±0.47 de	2.82	±0.05 cd	1.66	±0.47 a	2.84	±0.13 cd	2.77	±0.02 bc	2.31	±0.07 b	3.35	±0.22 e
	b*	11.1	±0.3 a	12.1	±0.1 c	12.2	±0.1 c	10.8	±0.4 a	11.6	±0.2 b	12.2	±0.1 c	12.2	±0.2 c	11.9	±0.1 bc
Whole Ground Seeds	L*	49.8	±0.0 d	58.9	±0.0 f	46.0	±0.0 c	54.0	±0.1 e	45.8	±0.1 c	41.7	±0.0 a	45.7	±0.1 d	45.7	±0.1 b
	a*	1.81	±0.02 c	3.57	±0.03 f	2.26	±0.03 e	1.35	±0.05 a	2.31	±0.03 e	2.28	±0.07 a	1.47	±0.02 b	1.97	±0.07 d
	b*	11.0	±0.0 f	15.4	±0.0 h	10.3	±0.0 d	10.4	±0.0 e	9.9	±0.0 c	9.5	±0.0 b	11.5	±0.0 g	9.2	±0.0 a

Mean ± SD (n = 3). Values followed by the same letter in the same row are not significantly different (*p <* 0.05).

**Table 3:** Comparison of the total color difference (ΔE*) between commercial chia seeds and whole ground seeds.

ΔE*	Chia Origin									
		Seeds	Bolivian		Argentina	Chile	Ecuador	Peru	Mexico	Paraguay
	Whole Ground		Dark	White						
Chia Origin	Bolivian	Dark	0	19.7	3.7	2.6	6.3	4.2	3.6	7.3
		White	10.3	0	22.9	17.2	25.7	23.5	23.0	26.6
	Argentina		3.9	14.3	0	6.1	2.8	0.6	0.5	3.8
	Chile		4.2	7.4	8.1	0	8.7	6.7	6.1	9.8
	Ecuador		4.3	14.3	0.5	8.2	0	2.3	2.8	1.1
	Peru		8.2	18.2	4.3	12.3	4.1	0	0.7	3.2
	Mexico		4.2	14.0	1.5	8.4	1.9	4.5	0	3.8
	Paraguay		4.5	14.7	1.2	8.4	0.8	3.9	2.4	0

**Table 4:** Proximal composition of commercial chia seeds.

Component (g/100 g d.m.)	Bolivian				Argentina		Chile		Ecuador		Peru		Mexico		Paraguay	
	Dark		White													
Moisture	8.62	±0.13 g	7.09	±0.11 a	8.20	±0.10 d	9.15	±0.21 h	7.89	±0.15 c	8.46	±0.08 f	8.35	±0.08 e	7.13	±0.10 b
Protein	21.4	±0.4 d	24.3	±0.0 e	20.3	±0.1 c	22.2	±0.1 d	17.7	±0.1 a	19.4	±0.0 b	20.2	±0.0 c	20.4	±0.1 c
Lipids	32.1	±0.8 bc	29.0	±0.0 a	30.6	±0.7 ab	34.9	±0.6 d	30.8	±0.1 ab	30.5	±0.6 ab	30.1	±0.5 ab	34.5	±0.2 cd
Ash	4.48	±0.21 a	4.34	±0.03 a	4.43	±0.28 a	4.79	±0.27 a	4.31	±0.24 a	4.79	±0.03 a	4.47	±0.32 a	4.34	±0.22 a

Mean ± SD (n = 3). Values followed by the same letter in the same row are not significantly different (*p <* 0.05).

**Table 5:** Dietary fiber of commercial chia seeds.

Component (g/100 g d.m.)	Bolivian				Argentina		Chile		Ecuador		Peru		Mexico		Paraguay	
	Dark		White													
Total Dietary Fiber *	20.0	±0.8 de	18.5	±1.5 bc	18.4	±1.3 cd	21.1	±0.1 ef	22.4	±0.4 f	26.0	±0.3 e	17.0	±0.6 ab	16.8	±0.2 a
Soluble (S)	4.55	±0.01 bc	5.41	±1.20 c	2.80	±0.61 a	4.21	±0.65 abc	3.50	±0.80 ab	5.46	±1.50 d	4.25	±0.10 abc	4.51	±0.05 abc
Insoluble (I)	15.5	±0.0 c	13.1	±1.2 b	15.6	±0.6 c	16.9	±0.6 cd	18.8	±0.9 d	20.5	±1.5 a	12.8	±0.1 b	12.3	±0.1 b
Ratio (S)/(I)	1:4.4		1:2.5		1:5.6		1:4.1		1:5.5		1:3.8		1:3.0		1:2.7	

Mean ± SD (n = 3). Values followed by the same letter in the same row are not significantly different (*p <* 0.05), * Adequate intake (AI) 25 g per day in adult ≥ 18 years [43].

**Table 6:** Amino acid composition of commercial chia seeds from different origin.

Amino Acid Composition g/100 g Seeds	Bolivian				Argentina		Chile		Ecuador		Peru		Mexico		Paraguay	
	Dark		White													
Non-essential																
Alanine	1.21	±0.01 d	1.09	±0.04 c	0.969	±0.011 b	1.10	±0.06 c	0.897	±0.002 a	0.942	±0.020 ab	1.11	±0.01 c	1.04	±0.03 c
Arginine	2.62	±0.05 e	2.18	±0.15 d	1.98	±0.04 bc	2.16	±0.11 cd	1.78	±0.08 a	1.92	±0.01 ab	2.28	±0.06 d	2.16	±0.11 cd
Aspartic acid	2.25	±0.02 f	1.93	±0.08 de	1.75	±0.01 bc	1.83	±0.11 cd	1.47	±0.02 a	1.65	±0.01 b	2.03	±0.01 e	1.81	±0.06 cd
Glutamic acid	4.41	±0.10 c	3.78	±0.23 b	3.40	±0.04 a	3.76	±0.28 b	3.17	±0.01 a	3.41	±0.01 a	3.98	±0.06 b	3.83	±0.15 b
Glycine	1.14	±0.01 f	1.03	±0.03 de	0.955	±0.029 bc	0.967	±0.047 bc	0.880	±0.011 a	0.937	±0.004 b	1.06	±0.01 e	1.00	±0.01 cd
Proline	0.944	±0.074 d	0.819	±0.047 bc	0.821	±0.044 abc	0.809	±0.036 bc	0.752	±0.041 a	0.875	±0.069 bcd	0.933	±0.037 cd	0.851	±0.013 abcd
Serine	1.37	±0.07 e	1.21	±0.04 d	1.09	±0.02 bc	1.17	±0.06 cd	0.969	±0.020 a	1.06	±0.00 ab	1.22	±0.04 d	1.12	±0.03 bcdc
Essential amino acid																
Tryptophan	0.333	±0.016 a	0.321	±0.001 a	0.268	±0.001 a	0.299	±0.001 a	0.253	±0.005 a	0.278	±0.016 a	0.331	±0.013 a	0.297	±0.020 a
Cysteine	0.497	±0.004 d	0.475	±0.004 cd	0.453	±0.002 bc	0.497	±0.009 d	0.422	±0.000 a	0.447	±0.023 ab	0.475	±0.021 cd	0.479	±0.002 cd
Methionine	0.671	±0.006 de	0.686	±0.025 e	0.610	±0.018 bc	0.646	±0.042 cde	0.527	±0.018 a	0.598	±0.004 b	0.637	±0.006 bcd	0.609	±0.004 bc
Histidine	0.673	±0.042 d	0.606	±0.023 c	0.525	±0.021 ab	0.583	±0.042 c	0.500	±0.008 a	0.524	±0.000 ab	0.603	±0.006 c	0.571	±0.008 bc
Isoluecine	0.847	±0.012 d	0.795	±0.044 cd	0.673	±0.023 ab	0.758	±0.071 c	0.604	±0.002 a	0.675	±0.018 ab	0.781	±0.003 cd	0.729	±0.023 bc
Leucine	1.60	±0.05 d	1.45	±0.08 c	1.29	±0.01 ab	1.42	±0.11 c	1.18	±0.00 a	1.27	±0.04 ab	1.47	±0.01 c	1.38	±0.04 bc
Lysine	1.17	±0.03 d	1.04	±0.04 c	0.952	±0.021 ab	1.04	±0.05 c	0.907	±0.008 a	0.951	±0.006 ab	1.08	±0.04 c	1.02	±0.06 bc
Phenylalanine	1.27	±0.01 d	1.12	±0.06 c	0.985	±0.049 ab	1.08	±0.08 bc	0.939	±0.038 a	1.01	±0.04 ab	1.16	±0.02 c	1.08	±0.03 bc
Threonine	0.896	±0.030 e	0.789	±0.015 cd	0.726	±0.004 b	0.762	±0.042 bc	0.628	±0.008 a	0.714	±0.025 b	0.837	±0.001 d	0.759	±0.024 bc
Tyrosine	0.878	±0.007 c	0.741	±0.048 ab	0.663	±0.054 a	0.734	±0.040 ab	0.647	±0.006 a	0.689	±0.035 a	0.806	±0.086 bc	0.748	±0.045 ab
Valine	1.16	±0.00 d	1.03	±0.06 c	0.939	±0.021 ab	1.00	±0.07 bc	0.859	±0.003 a	0.905	±0.009 a	1.06	±0.00 c	1.00	±0.02 bc
EAAs	9.98		9.04		8.08		8.81		7.46		8.06		9.22		8.67	

Mean ± SD (n = 3). Values followed by the same letter in the same row are not significantly different (*p <* 0.05); d.m. dry matter; EAAs, essential amino acids.

**Table 7:** Fatty acid composition of commercial chia seeds.

Parameter Fatty Acid		Units or Reference Values	Bolivian				Argentina		Chile		Ecuador		Peru		Mexico		Paraguay	
			Dark		White													
Palmitic acid	C16:0	g/100 g d.m.	2.11	±0.15 g	2.15	±0.25 h	1.89	±0.22 a	1.91	±0.12 b	2.10	±0.13 f	1.95	±0.11 c	2.02	±0.25 e	2.00	±0.14 d
Stearic acid	C18:0	g/100 g d.m.	0.94	±0.23 c	1.01	±0.18 f	0.84	±0.47 a	0.86	±0.04 b	0.95	±0.04 d	1.01	±0.06 f	1.05	±0.11 g	1.00	±0.11 e
Arachidic acid	C20:0	g/100 g d.m.	0.07	±0.00 b	0.08	±0.02 c	0.07	±0.00 b	0.06	±0.00 a	0.07	±0.00 b	0.08	±0.02 c	0.07	±0.05 b	0.08	±0.01 c
Ʃ SFA			3.12		3.24		2.80		2.83		3.12		3.04		3.14		3.08	
Palmitoleic acid	C16:1n7	g/100 g d.m.	0.05	±0.01 a	0.08	±0.01 d	0.06	±0.01 b	0.06	±0.04 b	0.07	±0.01 c	0.06	±0.01 b	0.07	±0.01 c	0.06	±0.01 b
Oleic acid	C18:1n9	g/100 g d.m.	1.74	±0.40 d	2.02	±0.21 g	1.50	±0.16 b	1.40	±0.03 a	1.55	±0.07 c	1.97	±0.23 f	2.67	±0.19 h	1.96	±0.10 e
Vaccenic acid	C18:1n7	g/100 g d.m.	0.14	±0.05 c	0.14	±0.00 c	0.13	±0.11 b	0.13	±0.08 b	0.14	±0.08 c	0.13	±0.09 b	0.12	±0.08 a	0.13	±0.06 b
Ʃ MUFA			1.93		2.24		1.69		1.59		1.76		2.16		2.86		2.15	
Linoleic acid (LA)	C18:2n6c	g/100 g d.m.	5.56	±0.25 e	5.66	±0.11 f	5.28	±0.30 c	4.88	±0.31 a	5.15	±0.34 b	5.50	±0.66 d	5.97	±0.13 g	5.56	±0.52 e
γ-Linolenic acid	C18:3n6	g/100 g d.m.	0.05	±0.01 a	0.06	±0.04 b	0.05	±0.01 a	0.05	±0.01 a	0.06	±0.00 b	0.06	±0.01 b	0.05	±0.02 a	0.05	±0.01 a
α-Linolenic acid (ALA)	C18:3n3	g/100 g d.m.	16.9	±1.1 c	15.6	±0.5 b	18.2	±0.8 g	18.4	±0.6 h	17.4	±0.0 d	18.1	±0.6 f	14.9	±0.2 a	18.0	±0.3 e
Ʃ PUFA			22.49		21.32		23.57		23.28		22.64		23.62		20.93		23.59	
LA/ALA	C18:2n6c/C18:3n3	g/g	1/3		1/2.8		1/3.5		1/3.8		1/3.4		1/3.3		1/2.5		1/3.2	
% of contribution of AI E% for LA ^a^	FAO	2.5 E%	13.2		13.5		12.6		11.6		12.3		13.1		14.2		13.2	
	EFSA	4 E%	8.27		8.42		8.27		7.26		7.66		8.18		8.88		8.27	
% of DRI of LA ^a^	EFSA	17 g/day (Male)	4.91		4.99		4.66		4.31		4.54		4.85		5.27		4.91	
		12 g/day (Female)	7.0		7.1		6.6		6.1		6.4		6.8		7.5		7.0	
% of contribution of AI E% for ALA ^a^	FAO/EFSA	0.5 E%	201		186		217		218		208		215		177		214	
% of DRI of ALA ^a^	EFSA	1.6 g/day (Male)	158		146		171		172		164		169		140		169	
		1.1 g/day (Female)	230		213		249		250		238		246		203		245	

Mean ± SD (n = 3). Values followed by the same letter in the same row are not significantly different (*p* < 0.05); Adequate intake (AI) contribution expressed in energy percentage (E%) for LA and ALA for adults (≥18 age) [43,44]; SFA (short fatty acid), MUFA (monounsaturated fatty acid), PUFA (polyunsaturated fatty acid) and DRI (Dietary Recommendation Intake); ^a^ Contribution based on limit intake chia seed (15 g/day) taking in account a 2200 Kcal diet [52].

**Table 8:** Phytic acid content and mineral composition of commercial chia seeds from different origins, mineral dietary reference intake contribution and their bioavailability prediction.

	Parameter		Units		Bolivian		Argentina	Chile	Ecuador	Peru	Mexico	Paraguay
					Dark	White						
	Ins *P*_6_		g/100 g d.m.		2.6 ± 0.0 d	1.5 ± 0.03 a	2.2 ± 0.04 c	2.6 ± 0.05 d	2.2 ± 0.07 c	1.9 ± 0.0 b	2.3 ± 0.06 c	2.2 ± 0.01 c
Macroelements	Na		mg/100 g d.m.		1.2 ± 1.4 a	1.0± 0.5 a	1.3 ± 0.1 a	1.0 ± 0.0 a	1.2 ± 0.0 a	1.4 ± 0.1 a	1.2 ± 0.6 a	0.8 ± 0.0 a
	Ca		mg/100 g d.m.		498.9 ± 0.1 ab	492.3 ± 0.3 ab	537.1 ± 0.1 abc	671.1 ± 0.2 c	481.5 ± 0.2 ab	613.4 ± 0.2 bc	530.9 ± 0.3 ab	460 ± 0.1 a
	P		mg/100 g d.m.		549.6 ± 0.4 d	393.9 ± 0.7 a	472.3 ± 0.5 bc	661.9 ± 0.4 e	482 ± 0.4 bcd	409.1 ± 0.8 ab	474.2 ± 1.1 bc	540.3 ± 0.7 cd
	Mg		mg/100 g d.m.		280.8 ± 0.5 abc	262.6 ± 0.2 ab	263.8 ± 0.3 ab	321.6 ± 0.3 d	301.2 ± 0.9 bcd	261.7 ± 0.3 a	250 ± 0.4 a	308.6 ± 0.2 cd
Microelements	Zn		mg/100 g d.m.		3.69 ± 0.00 e	4.83 ± 0.01 cd	2.69 ± 0.03 a	5.16 ± 0.01 f	3.69 ± 0.00 cd	3.55 ± 0.01 bc	3.37 ± 0.04 b	3.94 ± 0.01 d
	Fe		mg/100 g d.m.		7.79 ± 0.58 a	7.55 ± 0.25 a	6.12 ± 0.06 a	5.57 ± 0.13 a	5.74 ± 0.02 a	23.4 ± 0.97 b	4.23 ± 0.05 a	4.64 ± 0.00 a
	Mn		mg/100 g d.m.		2.06 ± 0.04 c	1.47 ± 0.01 b	2.49 ± 0.00 d	0.94 ± 0.00 a	3.95 ± 0.02 f	2.58 ± 0.01 d	2.96 ± 0.04 e	5.95 ± 0.01 g
	Cu		mg/100 g d.m.		1.07 ± 0.01 b	1.50 ± 0.01 cd	0.91 ± 0.01 a	1.58 ± 0.00 d	1.42 ± 0.01 cd	1.28 ± 0.00 c	0.86 ± 0.01 a	1.32 ± 0.02 c
	Al		mg/100 g d.m.		1.95 ± 0.37 a	2.15 ± 0.11 a	3.02 ± 0.01 a	0.59 ± 0.03 a	4.35 ± 0.39 a	41.15 ± 0.16 b	0.8 ± 0.02 a	0.25 ± 0.03 a
	S		mg/100 g d.m.		180 ± 1 abc	216 ± 1 d	175± 0 ab	223± 1 d	170 ± 1 a	194 ± 1 c	166 ± 0 a	190 ± 1 bc
Ratio	Ins P_6_/Ca < 0.24		mol/mol		0.37	0.22	0.29	0.27	0.32	0.22	0.30	0.35
	Ins P_6_/Fe < 1		mol/mol		32.9	20.2	35.6	46.1	37.9	8.0	52.6	47.8
	Ins P_6_/Zn < 15		mol/mol		81.4	37.1	94.9	58.3	69.1	61.3	77.3	65.8
Contribution to DRIs (%) ^b^	Na	DRIs ^a^ 1500		mg/day	4.7	4.9	5.4	6.7	4.8	6.1	5.3	4.6
	Ca	DRIs ^a^ 1000		mg/day	7.4	7.4	8.1	10.1	7.2	9.2	8.0	6.9
	P	DRIs ^a^ 550		mg/day	15.0	10.7	12.9	18.1	13.2	11.2	12.9	12.9
	Mg	DRIs ^a^ 350/300		mg/day	12.0/14.0	11.3/13.1	11.3/13.2	13.8/16.1	12.9/15.1	11.2/13.1	10.7/12.5	13.2/15.4
	Zn	DRIs ^a^ 11/8		mg/day	5.0/6.9	6.6/9.1	3.7/5.0	6.6/9.1	5.0/6.9	4.8/6.7	4.6/6.3	4.6/6.3
	Fe	DRIs ^a^ 11/7		mg/day	10.6/16.7	10.3/16.2	8.4/13.1	7.6/11.9	7.8/12.3	31.9/50.1	5.8/9.1	6.3/9.9
	Mn	DRIs ^a^ 2.3/1.8		mg/day	13.4/17.2	13.4/17.2	16.2/20.8	6.1/7.8	25.8/32.9	16.8/21.5	19.3/24.7	38.8/49.6
	Cu	DRIs ^a^ 1.6/1.3		mg/day	10.0/12.4	14.1/17.3	8.5/10.5	14.8/18.2	13.3/16.4	12/14.8	8.1/9.9	12.4/15.2

Mean ± SD (n = 3). Values followed by the same letter in the same row are not significantly different (*p* < 0.05). Ins*P*_6_: *Myo*-inositol phosphate. ^a^ DRIs: Dietary reference intakes: recommended dietary allowances and adequate intakes, elements (Male/Female). Life stage group: >18 years; [62]. ^b^ Contribution based on limited chia seed intake (15 g/day) by EFSA [52].

## Data Availability

The data used to support the findings of this study can be made available by the corresponding author upon request.
